# Formation of carbohydrate-functionalised polystyrene and glass slides and their analysis by MALDI-TOF MS

**DOI:** 10.3762/bjoc.8.86

**Published:** 2012-05-21

**Authors:** Martin J Weissenborn, Johannes W Wehner, Christopher J Gray, Robert Šardzík, Claire E Eyers, Thisbe K Lindhorst, Sabine L Flitsch

**Affiliations:** 1 School of Chemistry & Manchester Interdisciplinary Biocentre, The University of Manchester, 131 Princess Street, Manchester, M1 7DN, United Kingdom; 2 Otto Diels Institute of Organic Chemistry, Christiana Albertina University of Kiel, Otto-Hahn-Platz 3/4, 24098 Kiel, Germany

**Keywords:** carbohydrate array, conductive tape, MALDI-TOF MS, nonconductive surface, trityl-mediated adhesion

## Abstract

Glycans functionalised with hydrophobic trityl groups were synthesised and adsorbed onto polystyrene and glass slides in an array format. The adsorbed glycans could be analysed directly on these minimally conducting surfaces by MALDI-TOF mass spectrometry analysis after aluminium tape was attached to the underside of the slides. Furthermore, the trityl group appeared to act as an internal matrix and no additional matrix was necessary for the MS analysis. Thus, trityl groups can be used as simple hydrophobic, noncovalently linked anchors for ligands on surfaces and at the same time facilitate the in situ mass spectrometric analysis of such ligands.

## Introduction

Microarrays have become valuable tools in the high-throughput analysis of biological interactions and have promising applications for the development of diagnostic devices in clinical environments [1]. The initial success with DNA microarrays has prompted investigations into other biomolecular ligands, such as protein, peptide and carbohydrate arrays [2–3]. An important aspect of this field is the immobilisation of such ligands on solid array surfaces, which can be polymers, such as polystyrene, or glass or gold, i.a. [4–7]. The challenge for immobilisations is in the efficiency of coupling and analysis of the attached ligands to ensure quality control.

**Figure 1:**
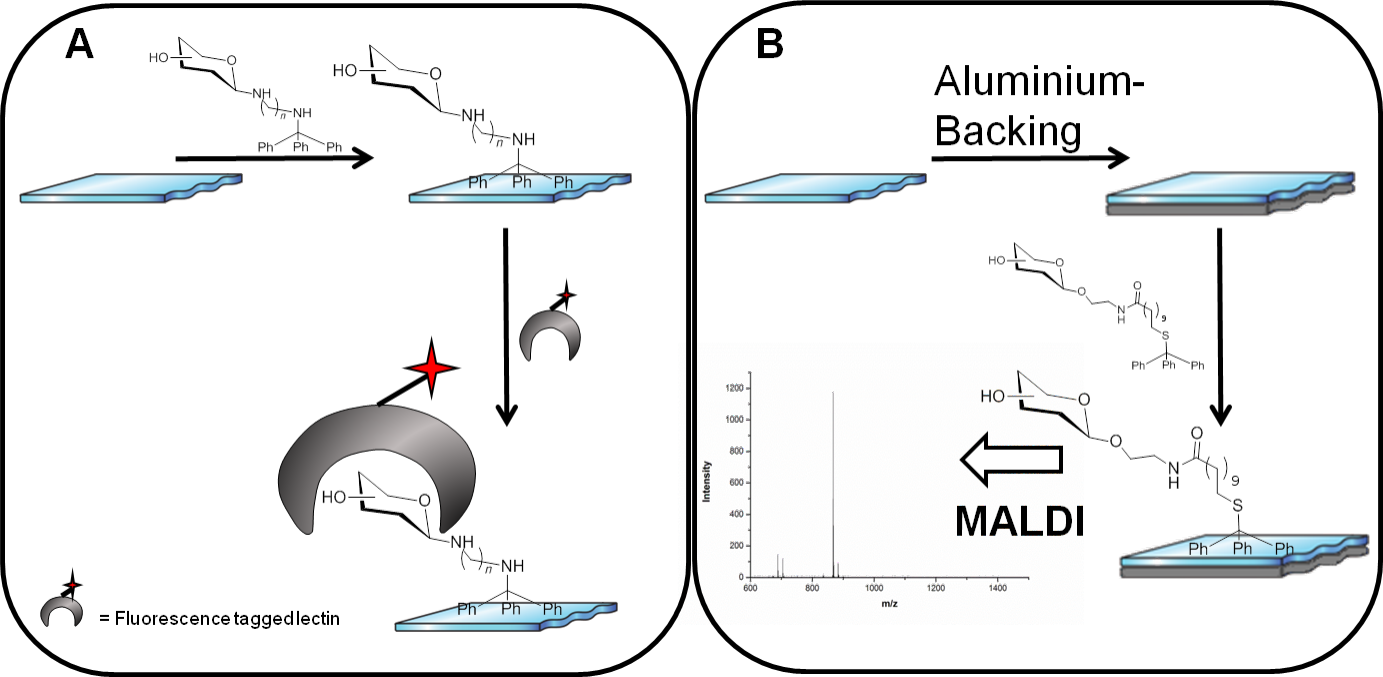
Carbohydrate arrays on polystyrene slides can be obtained by noncovalent immobilisation of tritylated saccharide derivatives. (A) Lectin-mediated analysis of carbohydrate arrays [7]. (B) The new concept of label-free MALDI-TOF MS analysis by aluminium-backing of polystyrene or glass slides.

Noncovalent attachment of biomolecules to hydrophobic surfaces has been used for a long time in ELISA assays and is attractive because no coupling reagents are required. However, it requires inherent hydrophobicity in the biomolecule or attachment of a hydrophobic tether. The latter has been used highly successfully by the Feizi group as part of the neoglycolipid array technology [8]. More recently, two groups [7,9] have reported the application of hydrophobic tethers for binding to polystyrene slides for glycan analysis. Initially, simple alkyl chains were used as tethers [9] but more recently, Wong and co-workers improved on this technology by using trityl-derived glycans, which are easily attached to glycans and were reported to bind strongly to polystyrene (Figure 1A) [7].

The attachment of glycans to the surface was generally confirmed indirectly by lectin binding, which severely limits the ligands that can be interrogated to those that can be detected by carbohydrate-binding proteins.

To overcome this limitation, we were interested in developing label-free methods for ligand detection on these polystyrene surfaces, and have investigated the use of matrix-assisted laser desorption/ionisation time-of-flight (MALDI-TOF) mass spectrometry (MS) analysis (Figure 1B), which has been highly successful on ligands immobilised on gold plates [10]. MALDI-TOF MS requires an electrically conducting surface and a matrix for analysis. The matrix is typically cocrystallised with the sample, which can lead to irregular surfaces, which can be a problem for reproducible analysis, especially when used in array format as a high-throughput tool. To avoid the use of such a matrix, we were interested in investigating trityl functionalisation, which has the potential for self-formation of a matrix (self-matrix) for MS analysis, at the same time as acting as a hydrophobic tether [11–13]. However, polystyrene has only minimal innate electrical conductivity and to our knowledge has never been used successfully, unmodified, as a target for MALDI-TOF MS analysis. Based on previous work one predicts that photoelectrons generated by UV laser irradiation are not dissipated by the polymeric surface. These photoelectrons distort the local electric field causing a significant loss in resolution of the analyte ions and a nonlinear shift in the mass-to-charge ratio [14].

Previous attempts to get around this issue have involved coating polymer or glass surfaces with a thin membrane of conductive material, such as gold, carbon or indium-tin oxide [15–17], or the addition of electron-accepting additives, such as methyl viologen dichloride hydrate [14]. The addition of electron-accepting additives, however, did not completely suppress the mass shifts observed during MALDI-TOF MS on low-conductivity supports. Additionally, glass slides coated with conductive material are expensive and limited in their utility [14].

In order to address these issues, we have investigated a simple and cheap method to enable MALDI-TOF MS analysis on minimally conductive supports by applying a commercially available aluminium tape to the underside of glass and polystyrene slides. This has allowed us to make standard microscope slides suitable for MALDI-TOF MS analysis.

## Results and Discussion

The hydrophobic trityl tethers chosen for our studies consisted of *S*-tritylated instead of *N*-tritylated groups, which were used previously [7]. This followed the idea of “orthogonal” surface functionalisation. Thus, a thiol group would make the tethers generally compatible with other platforms in our laboratories, such as through the formation of SAMs on gold [18] or by coupling into maleimide-functionalised surfaces in a chemoselective fashion [19].

**Scheme 1:**
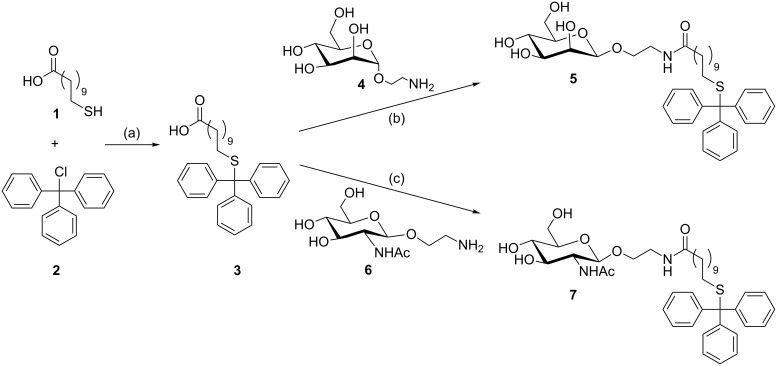
Synthesis of the tritylated compounds **3** [28], **5** and **7**: (a) dichloromethane, 2.5 h, rt, 98%, (b) HBTU/DIPEA, dry DMF, overnight, rt, 74%, (c) HATU/DIPEA, dry DMF, 3 h, rt, 71%.

For the initial studies two carbohydrate derivatives, **5** and **7**, were synthesised. The α-D-mannoside **5** would be useful in a bacterial adhesion inhibition assay against the bacterial lectin FimH [20–21]. The second glycoside **7** has been used previously for well-established enzymatic surface modifications [22]. Both these compounds can be synthesised by starting from commercially available 11-mercaptoundecanoic acid (**1**), which is tritylated with **2** in a straightforward synthesis, in 98% yield, following the procedure of Kovács et al. [23] (Scheme 1). The acid **3** was coupled with either of the aminoethyl glycosides **4** [24–26] or the GlcNAc derivative **6** [27], which were prepared according to literature procedures. For the coupling a combination of HBTU/DIPEA or HATU/DIPEA in dry DMF was applied yielding 74% of **5** and 71% of **7**.

The analysis of molecules on materials such as polystyrene by MALDI-TOF MS is difficult and irreproducible, largely due to their minimal electrical conductivity. To our knowledge, successful MS analysis on such surfaces has not been reported. In order to circumvent this issue, commercially available aluminium tape was applied to the underside of a polystyrene microscope slide.

**Figure 2:**
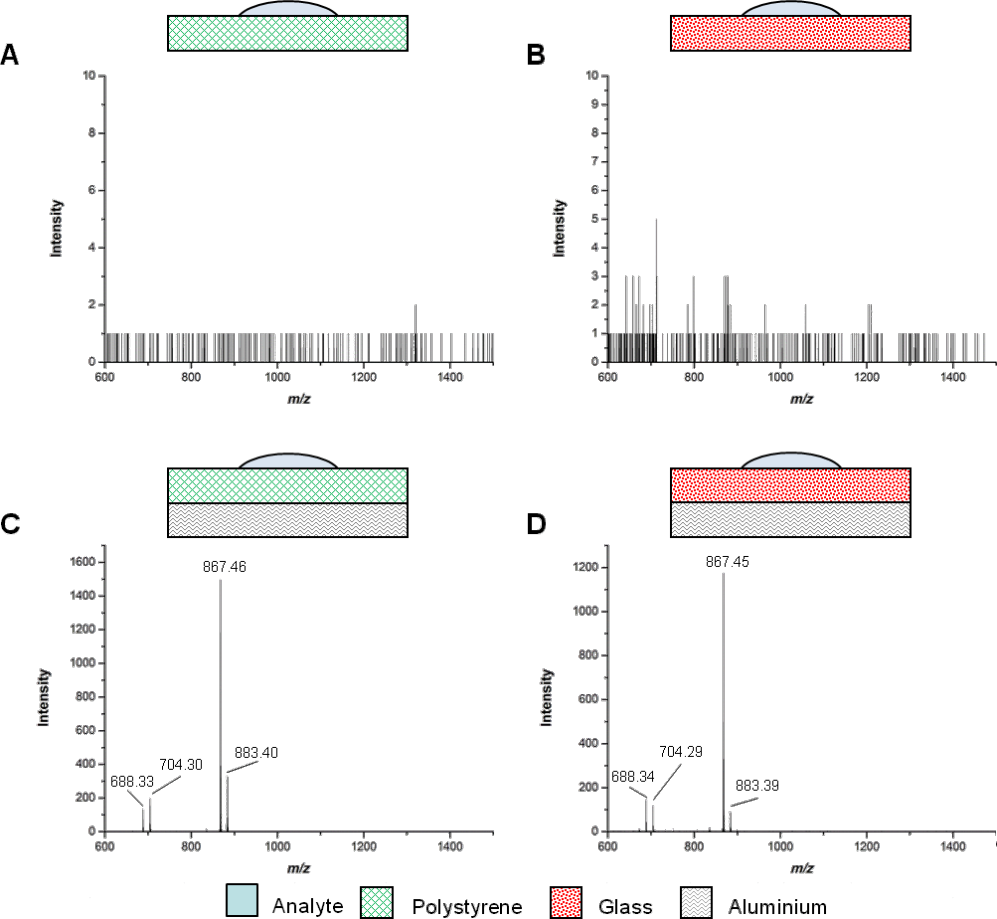
Comparison of the polystyrene and glass surfaces with and without aluminium backing by matrix-free MALDI-TOF MS analysis. Each spot contains 15 nmol Man–Trt (**5**): (A) polystyrene without aluminium; (B) glass without aluminium; (C) polystyrene with aluminium and (D) glass with aluminium. The peaks at *m*/*z* of 688.3, 704.3, 867.5 and 883.4 correspond to [**5** + Na]^+^, [**5** + K]^+^, [**8** + Na]^+^, and [**8** + K]^+^, respectively.

The tape significantly enhanced both the signal intensity and resolution of MALDI-TOF MS analysis of the Man–Trt compound **5** (Figure 2). Furthermore, we observed that analysis of the Man–Trt compound **5** could not only be performed in the absence of any additional matrix [11–12], but under these conditions a modest increase in mass-spectrometric resolution was also observed. Such self-matrix properties are very convenient, yielding more robust and reproducible analyses, and negating the need to search for “sweet spots”, as no crystal formation is required, in contrast to conventional MALDI-TOF MS analysis.

**Scheme 2:**
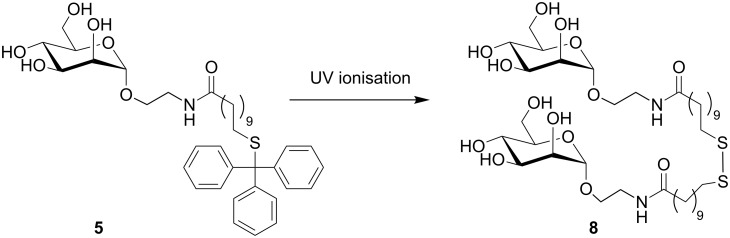
The Man–Trt compound **5** forms the disulfide **8** during UV ionisation in MALDI-TOF MS analysis.

Interestingly, both Na and K cation adducts of Man–Trt **5** and its disulfide **8** were observed (Scheme 2). The relative ratios of the monomer **5** and the disulfide **8** were found to be concentration-dependent in the analysis on stainless steel (Supporting Information File 1). It was observed that at 15 nmol, almost exclusively the disulfide **8** was detected. Conversely at 50 pmol only K^+^ and Na^+^ adducts of Man–Trt **5** were found.

We were interested to see whether the addition of the aluminium tape would also enhance signals on surfaces other than polystyrene, and we therefore assessed the influence of the conductive tape on analysis using glass slides, which are widely used in protein-, peptide- and glycoarrays [2,29–30]. As observed with the polystyrene slides, the resolution and signal intensity of MALDI-TOF MS analysis (Figure 2) was dramatically improved following application of the aluminium tape to the back of the glass slides.

**Figure 3:**
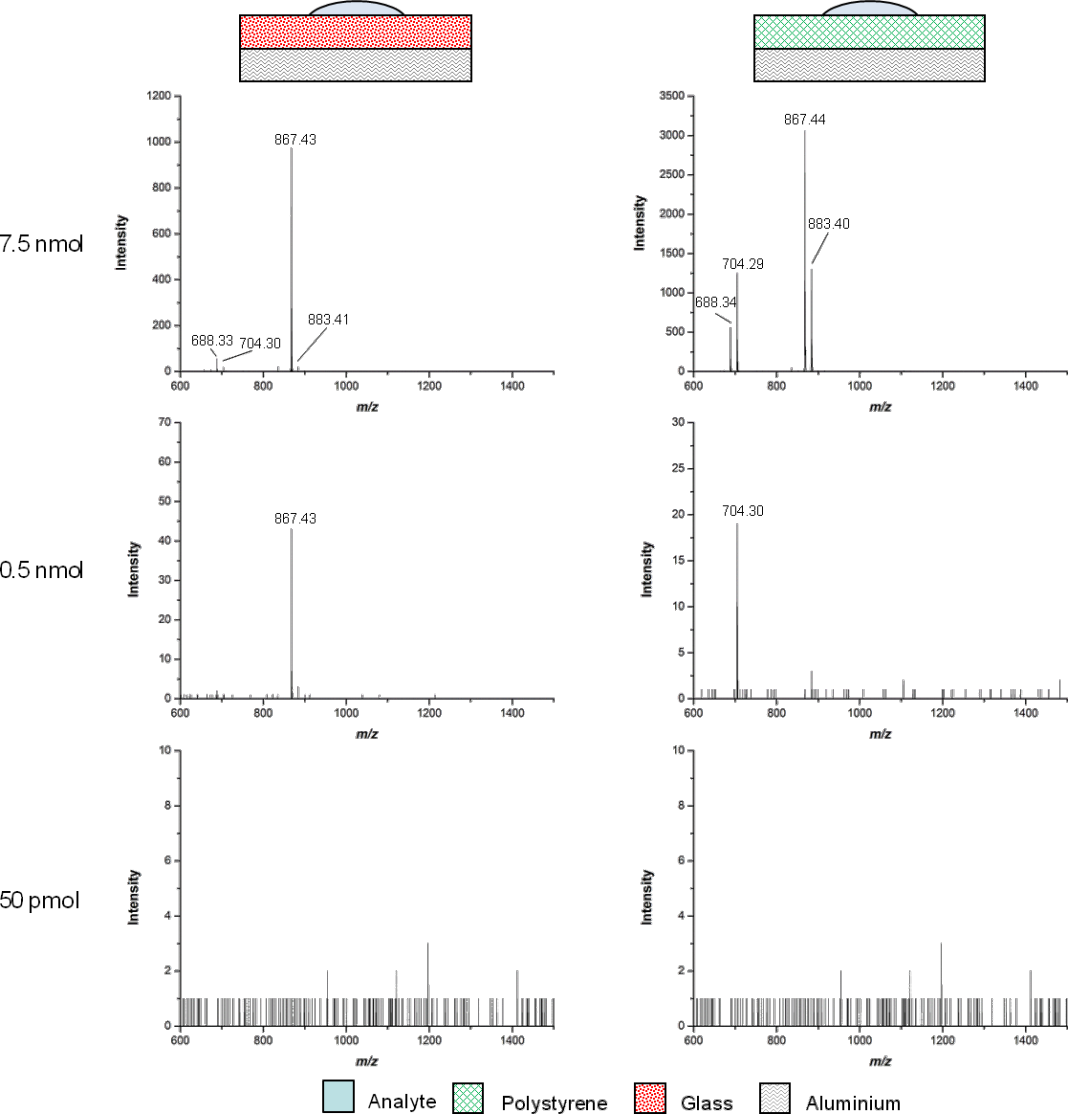
Limit-of-detection analysis of **5** on aluminium-backed glass and polystyrene slides. Both systems showed the MALDI-TOF MS detection limit at 0.5 nmol. The peaks at *m*/*z* of 688.3, 704.3, 867.4 and 883.4 correspond to [**5** + Na]^+^, [**5** + K]^+^, [**8** + Na]^+^, and [**8** + K]^+^, respectively.

Next, the limit of detection of MS analysis of aluminium-backed polystyrene and glass slides was compared (Figure 3), using dilutions of the analyte Man–Trt **5** from 15 nmol to 50 pmol. The resolution between the two supports was comparable and analysis showed that Man–Trt **5** could still be detected at a concentration of 0.5 nmol for aluminium-backed polystyrene and glass slides.

**Figure 4:**
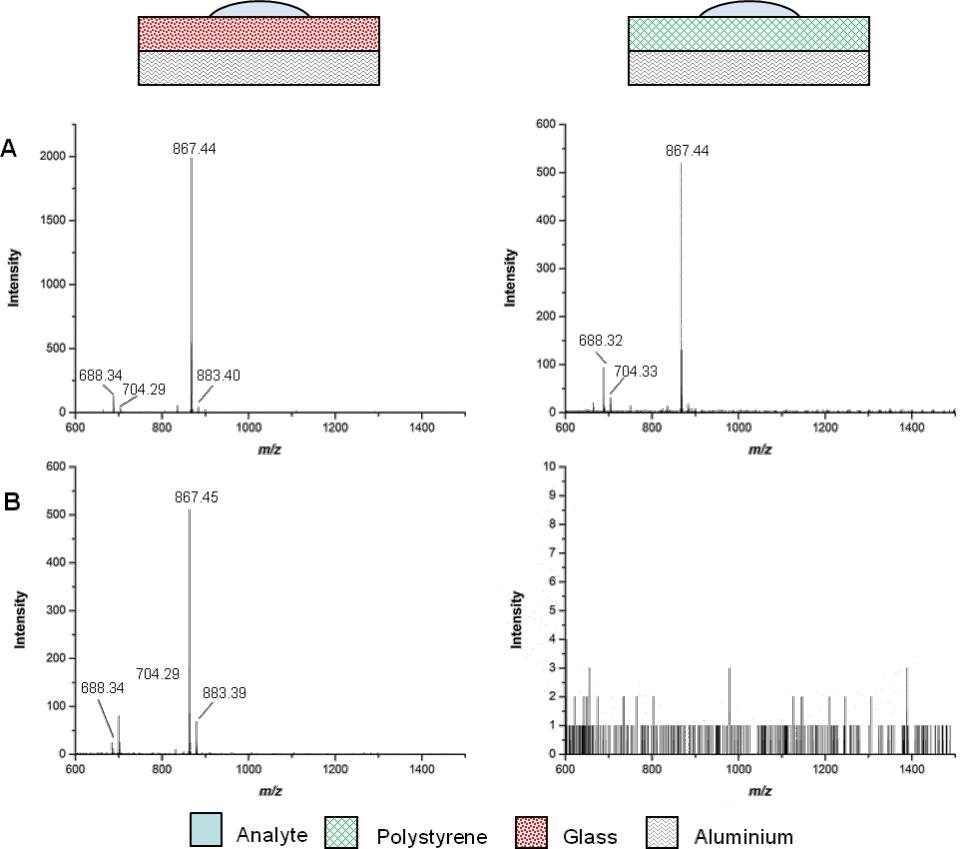
Comparison of MALDI-TOF MS spectra on the aluminium-backed polystyrene and glass slides after washing. (**A**) At 7.5 nmol following washing procedure 1 and (**B**) at 15 nmol following washing procedure 2.

After application of trityl samples, the polystyrene and glass slides were gently washed with 1 μL of water (washing procedure 1). Subsequent analysis by MALDI-TOF MS showed no noticeable change to the prewashed samples, confirming the trityl-group-mediated noncovalent adhesion of the ligands to the surfaces. On the other hand, much more rigorous washing under running distilled water (washing procedure 2) caused a significant reduction in signal on the polystyrene slide (Figure 4). Analysis of the glass slide, however, showed only a slight decrease in signal intensity even after three rigorous washes.

**Figure 5:**
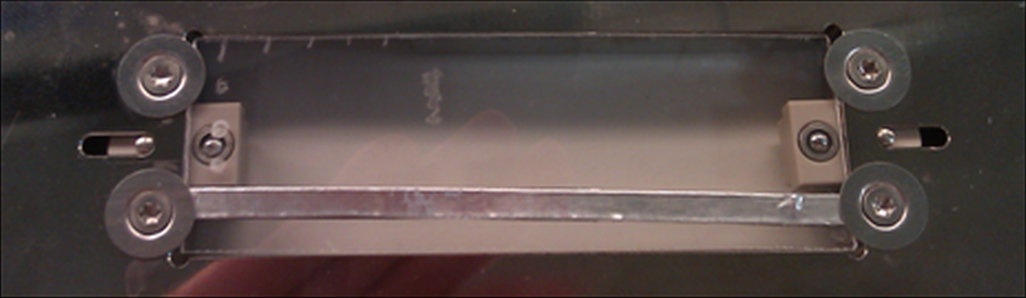
Photo of the aluminium strip on the back of the polystyrene support.

We were intrigued by the role of the aluminium backing and decided to investigate it in more detail. The aluminium backing was applied in three different ways: First, the entire underside of the slide was covered as in the previous experiments. Second, only a narrow strip of tape was applied to the back of the slide, making sure that the strip was in contact with the frame of the slide adapter (Figure 5). This configuration should still allow for efficient dissipation of any produced photoelectrons. Spots were analysed directly over and also next to the strip. The results showed similar intensity and resolution as for the fully aluminium-backed polystyrene slide (Supporting Information File 1, Figures S2 and S3). Third, only a small aluminium rectangle was attached to the back of the polystyrene slide, this time making sure that there was no electrical contact to the slide frame (Supporting Information File 1, Figures S4 and S5). In this last case very poor signals, both in resolution and intensity, were observed, which were analogous to the non-aluminium-backed polystyrene slide. Thus, contact of the tape to the slide adapter frame appears to be essential for good signal intensity, but it is not necessary to cover the slide fully.

Given that the MS analysis on the polystyrene was successful, we attempted an enzymatic galactosylation of GlcNAc–Trt **7** using bovine β-(1→4)-galactosyltransferase (β-(1→4)-GalT, EC 2.4.1.38) on the polystyrene slides. This enzymatic transformation has proven to be a very reproducible and robust reaction, which appears to proceed to completion on gold arrays [31] and is routinely performed in our laboratory. After treatment of slides containing GlcNAc–Trt **7** with the enzyme by using previously reported procedures [31], followed by washing procedure 1, neither product nor starting material could be observed by MALDI-TOF MS analysis. In fluorescence-assisted studies this problem is mostly overcome by blocking with nonfluorescent milk proteins or BSA; however, in MALDI-TOF MS analysis the blocking proteins would also be ionised and consequently quench the signal [32].

## Conclusion

Successful MALDI-TOF MS analysis on minimally conductive surfaces was achieved by application of aluminium tape. Polystyrene and glass surfaces were spotted with the analyte Man–Trt **5** and were analysed over a range of concentrations and after washing. This new technique enables the direct analysis of any noncovalent glycoarray on glass and polystyrene. So far, our attempts to study enzymatic reactions on the modified polystyrene surface have been unsuccessful and will require further investigation.

## Experimental

### General experimental section for the saccharide synthesis

Commercially available starting materials and reagents were used without further purification. Reactions requiring dry conditions were performed under an atmosphere of nitrogen. Anhydrous DMF was purchased.

2-Aminoethyl α-D-mannopyranoside (**4**) [24–26] and 2-aminoethyl 2-acetamido-2-deoxy-β-D-glucopyranoside (**6**) [27] were prepared according to the literature. Reactions were monitored by thin-layer chromatography using silica gel 60 GF_254_ on aluminium foil (Merck) with detection by UV light and charring with sulfuric acid in EtOH (10%). Preparative MPLC was performed on a Büchi apparatus using a LiChroprep Si 60 (40–60 mm, Merck) column for normal-phase silica-gel chromatography. Analytical HPLC was performed on a Merck Hitachi LaChrom L-7000 series apparatus with a LiChrospher 100 RP-8 (5 μm, Merck) column. ^1^H and ^13^C NMR spectra were recorded on a Bruker DRX-500 spectrometer. NMR spectra were calibrated with respect to the solvent peak. 2D NMR techniques (COSY, HSQC, HMBC) were used for full assignment of the spectra. ESI-MS measurements were performed on a Mariner ESI-TOF 5280 instrument (Applied Biosystems). High-resolution mass spectra (HRMS) were obtained with the Waters Micromass LCT-TOF mass spectrometer. MALDI-TOF mass spectra were recorded on a Bruker Biflex-III 19 kV instrument with Cl-CCA (4-chloro-α-cyanocinnamic acid) or DHB (2,5-dihydroxybenzoic acid) as matrix. Optical rotation was measured on a Perkin-Elmer polarimeter 341 (Na-D-line: 589 nm, length of cell 1 dm). IR spectra were recorded on a Perkin-Elmer Paragon 1000 FTIR spectrometer. For sample preparation a Golden Gate diamond ATR unit with a sapphire stamp was used.

#### 11-Tritylsulfanylundecanoic acid (**3**) [28]

Chlorotriphenylmethane (**2**, 2.53 g, 9.07 mmol) was dissolved in dichloromethane. 11-Mercaptoundecanoic acid (**1**, 2.00 g, 9.15 mmol) dissolved in dichloromethane (60 mL) was added dropwise over 1 h. The reaction mixture was stirred for 1.5 h at ambient temperature until TLC (cyclohexane/ethyl acetate 3:1) indicated no further conversion. The reaction mixture was washed with H_2_O (50 mL), the organic layer was dried over MgSO_4_, and the solvent was removed under reduced pressure. The crude product was purified by MPLC (100 g silica column, A: cyclohexane, B: ethyl acetate, A: 90% → 40%, 120 min) yielding **3** (4.10 g, 8.90 mmol, 98%) as a colourless solid.

*R*_f_ 0.61 (methanol/dichloromethane 3:18); mp 80–82 °C; HPLC *t*_R_ 7.31 min (A = water, B = methanol, A: 20%, 10 min, 1.2 mL/min); ^1^H NMR (500 MHz, CD_3_OD, 300 K) δ 7.38 (m_c_, 6H, H_aryl,Trt_), 7.25 (m_c_, 6H, H_aryl,Trt_), 7.19 (m_c_, 3H, H_aryl,Trt_), 2.18 (t, ^3^*J* = 7.6 Hz, 2H, HO(O)C*CH_2_*CH_2_), 2.11 (t, ^3^*J* = 7.4 Hz, 2H, *CH_2_*STrt), 1.59 (q, ^3^*J* = 7.9 Hz, ^3^*J* = 7.0 Hz, 2H, HO(O)CCH_2_*CH_2_*), 1.37–1.09 (m, 14H, CH_2_*CH_2_*CH_2_) ppm; ^13^C NMR (125 MHz, CD_3_OD, 300 K) δ 179.3 (*C*(O)OH), 146.5 (3 C_aryl,Trt_), 130.8 (6 CH_aryl,Trt_), 128.8 (6 CH_aryl,Trt_), 127.6 (3 CH_aryl,Trt_), 67.6 (C_quart,Trt_), 36.5 (HO(O)C*CH_2_*CH_2_), 32.9 (*CH_2_*STrt), 30.5, 30.4, 30.4, 30.3, 30.1, 30.0, 29.7 (7 CH_2_*CH_2_*CH_2_), 26.9 (HN(O)CCH_2_*CH_2_*) ppm; MALDI-TOF MS (DHB) *m*/*z*: 483.13 [M + Na]^+^, 499.10 [M + K]^+^; HRMS–ESI (*m*/*z)*: [M + Na]^+^ calcd for C_30_H_36_NNaO_2_S, 483.2328; found, 483.2343; IR (ATR–IR) 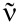: 3384, 3189, 3056, 2923, 2850, 1651, 1594, 1489, 1444, 1419, 1032, 770, 740, 695, 674, 621 cm^−1^.

#### 2-((11-Tritylsulfanylundecanoyl)amino)ethyl α-D-mannopyranoside (**5**)

11-Tritylsulfanylundecanoic acid (**3**, 750 mg, 1.63 mmol) and HBTU (743 mg, 1.96 mmol) were dried for 2 h under vacuum, and then dry DMF (5 mL) and DIPEA (400 μL, 2.33 mmol) were added, and the mixture was stirred for 20 min under a nitrogen atmosphere at ambient temperature. Simultaneously, in a different reaction vessel aminoethyl mannoside **4** (438 mg, 1.96 mmol) was dried for 2 h under vacuum, and then dissolved in dry DMF (5 mL), and DIPEA (160 μL, 931 μmol) was added. The mixture was stirred for 20 min under a nitrogen atmosphere at ambient temperature. The reaction mixture with the preactivated 11-tritylsulfanylundecanoic acid (**3**) was cooled to 0 °C, the solution of mannoside **4** was added and the resulting mixture was stirred under a nitrogen atmosphere at ambient temperature overnight. All volatile compounds were removed under reduced pressure and the crude product was subjected to MPLC (150 g silica column, A: dichloromethane, B: methanol, A: 99% → 90%, 120 min) and another round of MPLC (125 g silica column, A: ethyl acetate, B: methanol, A: 99% → 90%, 120 min) yielding **5** (808 mg, 1.21 mmol, 74%) as a colourless foam.

*R*_f_ 0.16 (methanol/dichloromethane, 1:9); HPLC *t*_R_ = 5.49 min (A = water, B = methanol, A: 20%, 10 min, 1.2 mL/min); 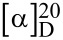 +23.7 (*c* 0.5, MeOH); ^1^H NMR (500 MHz, CD_3_OD, 300 K) δ 7.38 (m_c_, 6H, H_aryl,Trt_), 7.27 (m_c_, 6H, H_aryl,Trt_), 7.20 (dt, ^3^*J* = 7.3 Hz, ^4^*J* = 1.3 Hz, 3H, H_aryl,Trt_), 4.76 (d, ^3^*J* = 1.7 Hz, 1H, H1_Man_), 3.83 (dd, ^2^*J* = 11.6 Hz, ^3^*J* = 2.3 Hz, 1H, H6a_Man_), 3.80 (dd, ^3^*J* = 1.7 Hz, ^3^*J* = 3.3 Hz, 1H, H2_Man_), 3.77–3.67 (m, 3H, O*CH*HCH_2_NH, H3_Man_, H6b_Man_), 3.60 (dd~t, ^3^*J* = 9.5 Hz, 1H, H4_Man_), 3.56–3.50 (m, 2H, H5_Man_, O*C*H*H*CH_2_NH), 3.41 (ddd, ^2^*J* = 14.0 Hz, ^3^*J* = 6.3 Hz, ^3^*J* = 4.6 Hz, 1H, O*C*H_2_C*H*HNH), 3.35 (ddd, ^2^*J* = 14.0 Hz, ^3^*J* = 6.7 Hz, ^3^*J* = 4.7 Hz, 1H, O*C*H_2_CH*H*NH), 2.19 (t, ^3^*J* = 7.6 Hz, 2H, HN(O)C*CH_2_*CH_2_), 2.12 (t, ^3^*J* = 7.4 Hz, 2H, *CH_2_*STrt), 1.59 (q, ^3^*J* = 7.6 Hz, ^3^*J* = 7.3 Hz, 2H, HN(O)CCH_2_*CH_2_*), 1.38–1.10 (m, 14H, 7 CH_2_*CH_2_*CH_2_) ppm; ^13^C NMR (125 MHz, CD_3_OD, 300 K) δ 176.5 (*C*(O)NH), 146.5 (3 C_aryl,Trt_), 130.8 (6 CH_aryl,Trt_), 128.8 (6 CH_aryl,Trt_), 127.7 (3 CH_aryl,Trt_), 101.7 (C1_Man_), 74.8 (C5_Man_), 72.6 (C3_Man_), 72.1 (C2_Man_), 68.6 (C4_Man_), 67.3 (C_quart,Trt_), 67.3 (O*CH_2_*CH_2_NH), 62.9 (C6_Man_), 40.2 (OCH_2_*CH_2_*NH), 37.1 (HN(O)C*CH_2_*CH_2_), 32.9 (*CH_2_*STrt), 30.5, 30.4, 30.4, 30.3, 30.1, 30.0, 29.7 (7 CH_2_*CH_2_*CH_2_), 27.0 (HN(O)CCH_2_*CH_2_*) ppm; MALDI-TOF MS (DHB) *m*/z: 688.11 [M + Na]^+^, 704.08 [M + K]^+^; HRMS–ESI (*m*/*z*): [M + Na]^+^ calcd for C_40_H_54_N_2_NaO_7_S, 729.3544; found, 729.3506; IR (ATR–IR) 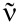: 3293, 2923, 2852, 1645, 1548, 1488, 1443, 1253, 1132, 1057, 1031, 975, 810, 741, 697, 676, 616 cm^−1^.

#### 2-((11-Tritylsulfanylundecanoyl)amino)ethyl 2-acetamido-2-deoxy-β-D-glucopyranoside (**7**)

11-Tritylsulfanylundecanoic acid (**3**, 18.2 mg, 39.5 μmol) and HATU (30.0 mg, 79.0 μmol) were dried for 1 h under vacuum. Then, dry DMF (2 mL) and DIPEA (7.00 μL, 40.9 μmol) were added, and the mixture was stirred for 20 min under a nitrogen atmosphere at ambient temperature. Simultaneously, in a different reaction vessel 2-aminoethyl 2-acetamido-2-deoxy-β-D-glucopyranoside (**6**, 11.5 mg, 43.5 μmol) was dried for 1 h under vacuum and dissolved in dry DMF (1 mL), and then DIPEA (7.00 μL, 40.9 μmol) was added. The mixture was stirred for 20 min under a nitrogen atmosphere at ambient temperature. The solution of **6** in dry DMF was added to the preactivated 11-tritylsulfanylundecanoic acid (**3**) and it was stirred under a nitrogen atmosphere at ambient temperature for 3 h. All volatile compounds were removed under reduced pressure and the crude product was subjected to MPLC (50 g silica column, A: dichloromethane, B: methanol, A: 99% → 85%, 180 min) yielding **7** (19.7 mg, 27.9 μmol, 71%) as a colourless lyophylisate.

*R*_f_ 0.21 (methanol/dichloromethane, 3:18); HPLC *t*_R_ 5.44 min (A = water, B = methanol, A: 20%, 10 min, 1.2 mL/min); 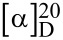 *−*1.6 (*c* 0.1, methanol); ^1^H NMR (500 MHz, CD_3_OD, 300 K) δ 7.39 (m_c_, 6H, H_aryl,Trt_), 7.28 (m_c_, 6H, H_aryl,Trt_), 7.21 (m_c_, 3H, H_aryl,Trt_), 4.39 (d, ^3^*J* = 8.4 Hz, 1H, H1_GlcNAc_), 3.88 (dd, ^2^*J* = 11.8 Hz, ^3^*J* = 2.2 Hz, 1H, H6a_GlcNAc_), 3.82 (ddd, ^2^*J* = 10.6 Hz, ^3^*J* = 6.7 Hz, ^3^*J* = 4.5 Hz, 1H, O*CH*HCH_2_NH), 3.67 (dd, ^2^*J* = 11.8 Hz, ^3^*J* = 5.8 Hz, 1H, H6b_GlcNAc_), 3.67–3.59 (m, 2H, H2_GlcNAc_, O*C*H*H*CH_2_NH), 3.43 (dd, ^3^*J* = 10.4 Hz, ^3^*J* = 8.3 Hz, 1H, H3_GlcNAc_), 3.40–3.36 (m, 1H, OCH_2_*CH*HNH), 3.40–3.26 (m, 3H, OCH_2_*C*H*H*NH, H4_GlcNAc_, H5_GlcNAc_), 2.18 (t, ^3^*J* = 7.6 Hz, 2H, HN(O)C*CH_2_*CH_2_), 2.12 (t, ^3^*J* = 7.4 Hz, 2H, *CH_2_*STrt), 1.98 (s, 3H, NHAc), 1.59 (m, 2H, HN(O)CCH_2_*CH_2_*), 1.38–1.10 (m, 14H, 7 CH_2_*CH_2_*CH_2_) ppm; ^13^C NMR (125 MHz, CD_3_OD, 300 K) δ 176.4 (HN*C*(O)CH_2_), 173.9 (HN*C*(O)CH_3_), 146.5 (3 C_aryl,Trt_), 130.8 (6 CH_aryl,Trt_), 128.8 (6 CH_aryl,Trt_), 127.7 (3 CH_aryl,Trt_), 102.9 (C1_GlcNAc_), 78.0 (C5_GlcNAc_), 76.1 (C3_GlcNAc_), 72.1 (C4_GlcNAc_), 69.2 (O*CH_2_*CH_2_NH), 67.3 (C_quart,Trt_), 62.8 (C6_GlcNAc_), 57.3 (C2_GlcNAc_), 40.6 (OCH_2_*CH_2_*NH), 37.1 (HN(O)C*CH_2_*CH_2_), 32.9 (*CH_2_*STrt), 30.5, 30.4, 30.4, 30.3, 30.1, 30.0, 29.7 (7 CH_2_*CH_2_*CH_2_), 27.0 (HN(O)CCH_2_*CH_2_*), 23.0 (HNC(O)*CH_3_*) ppm; MALDI-TOF MS (DHB) *m*/*z*: 729.48 [M + Na]^+^, 745.45 [M + K]^+^; HRMS–ESI (*m*/*z)*: [M + Na]^+^ calcd for C_40_H_54_N_2_NaO_7_S, 729.3544; found, 729.350; IR (ATR–IR) 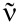: 3270, 2924, 2852, 1640, 1549, 1488, 1443, 1373, 1156, 1109, 1080, 1033, 896, 742, 698, 616 cm^−1^.

### Array washing

#### Washing procedure 1

Distilled water (1 μL) was spotted over the dried analyte spot and was subsequently drawn back up with the pipette. This was repeated three times. The slides were then allowed to dry under atmospheric conditions.

#### Washing procedure 2

The MALDI target slide was washed with cool distilled water (making sure the spot was not directly under the tap) for 6 s at a flow rate of 3 L/min and then dried under a stream of nitrogen.

### MALDI-TOF MS analysis of tritylated compounds

Unless otherwise stated all MALDI-TOF MS experiments were carried out on an Ultraflex II instrument (Bruker Daltonics, USA) in positive reflectron mode in the absence of a matrix. Spectra were acquired over the mass range 600–2500 *m*/*z* with 500 shots (57% laser energy) per spectrum and a laser firing rate of 200 Hz. Data were processed and analysed with FlexAnalysis software (Bruker Daltonics, USA) by using the default integration settings. Smoothing and baseline subtraction was performed on each spectrum by using the default settings in FlexAnalysis. Calibration was either performed before the analysis on the Ultraflex II instrument or afterwards in FlexAnalysis by using Man–Trt **5** as an internal calibrant for polystyrene and glass slides and a tryptic digest of Qcal protein as the calibrant for the steel target [33].

Polystyrene slides were manufactured by Goodfellows, U.K., and standard glass microscope slides, purchased from Yancheng Huida Medical Instruments Co., China, were used. Conductive aluminium tape was purchased from Farnell, U.K., and attached to the back of the nonconductive polystyrene and glass slides. The slides were mounted on to MTP Slide-Adapter II (Bruker) for analysis. Tritylated sugar in methanol (0.5 µL) was applied to the surface and the solvent was allowed to evaporate under atmospheric conditions. Unless otherwise stated, the spots were washed by following procedure 1.

## Supporting Information

File 1 Enzyme expression and MALDI MS spectra.
