# Supplementary material for: Formation of carbohydrate-functionalised polystyrene and glass slides and their analysis by MALDI-TOF MS
Source: Beilstein J Org Chem. 2012 May 21;8:753–62. doi: 10.3762/bjoc.8.86 (PMC3388864; doi:10.3762/bjoc.8.86)
Supplement: File 1 — Enzyme expression and MALDI MS spectra. [file Beilstein_J_Org_Chem-08-753-s001.pdf]

Supporting Information

for

Formation of carbohydrate-functionalised polystyrene and glass slides and their analysis by MALDI-TOF MS

Martin J. Weissenborn[1], Johannes W. Wehner[2], Christopher J. Gray[1], Robert Šardzík[1], Claire E. Eyers*[1], Thisbe K. Lindhorst*[2] and Sabine L. Flitsch*[1]

Address: [1]School of Chemistry & Manchester Interdisciplinary Biocentre, The University of Manchester, 131 Princess Street, Manchester, M1 7DN, United Kingdom and [2]Otto Diels Institute of Organic Chemistry, Christiana Albertina University of Kiel, Otto-Hahn-Platz 3/4, 24098 Kiel, Germany

Email: Sabine L. Flitsch* - sabine.flitsch@manchester.ac.uk; Thisbe K. Lindhorst* - tklind@oc.uni-kiel.de; Claire E. Eyers* - Claire.eyers@manchester.ac.uk

* Corresponding author

Enzyme expression and MALDI MS spectra

Table of contents

1. GalT1 enzyme S2
2. MALDI-TOF mass spectra S4

1. GalT1 enzyme

Subcloning of wild-type bovine GalT1 from Pichia expression vector into pET30a for expression in *E. coli*

The *P. pastoris* expression vector (pGAPz-GalT1) containing the GalT1 gene, which was used as template DNA, was kindly provided by Dr. Dubravko Rendić from the University of Natural Resources and Applied Life Sciences in Vienna.

Amplification of the plasmid was performed after transformation of *E. coli* TOP10F competent cells. The purified plasmid was used as a template to amplify the soluble region of the GalT1 gene by PCR using the primer pair EcGalT1-Fw/EcGalT1-Rv (EcGalT1-Fw: AAG AAT TCC TGC GAG GGG TCG CAC CGC CGC CGC CTT TGC AGA ACT CTT CC; EcGalT1-Rv: AAA AAA AGC GGC CGC CTA GCT CGG CGT CCC GAT GTC CAC TGT GAT TTT GG). The PCR product and pET30a vector were subjected to endonuclease treatment using EcoRI and NotI followed by ligation using T4 DNA ligase and subsequently dephosphorylated (by Calf Intestinal Alkaline Phosphatase). BL21(DE3) cells were transformed with the resulting plasmid construct for protein expression.

Expression, purification and refolding of the bovine GalT1

After transformation of *E. coli* (BL21 (DE3) with pET30a-GalT1, a single colony was used to inoculate 5 mL of LB medium containing kanamycin (50 µg/mL), and the culture was incubated at 37 °C/250 rpm. The overnight culture was used to inoculate 400 mL of fresh LB medium containing kanamycin (50 µg/mL). Cells were grown at 37 °C/250 rpm to an OD600 of 0.6, and protein expression was subsequently induced by the addition of IPTG (1 mM). The culture was further incubated at 37 °C/250 rpm for 6 h. Cells were harvested (5000 g, 10 min, 4 °C) and resuspended in 10 mL lysis buffer (50 mM Tris/HCl, 500 mM NaCl, 1% Triton X-100, 0.5 mM PMSF, pH 8.5). Lysis was performed by sonication (30 min, 10s on/10s off-cycles) followed by centrifugation (30000 g, 30 min, 4 °C). The pellet was

resuspended in binding buffer (50 mM Tris/HCl, 500 mM NaCl, 6 M urea, 10 mM imidazole, pH 8.5) and incubated overnight on a rocker at 4 °C in order to solubilize the enzyme from inclusion bodies. After centrifugation (30000 g, 30 min, 4 °C) the supernatant was filtered through a 0.2 µm filter and loaded onto a 5 mL HisTrap column pre-equilibrated with binding buffer. After washing of the column with 50 mL of loading buffer containing 6 M urea, the enzyme was eluted from the column by using 50 mM Tris/HCl, 500 mM NaCl, 6 M urea, 5000 mM Imidazole, pH 8.5. Eluted fractions containing protein were pooled and added dropwise at 4 °C under constant stirring to an excess of 100 mL refolding buffer (50 mM Tris/HCl, 5000 mM NaCl, 1% Triton X-100, 8 mM GSH, 1 mM GSSG, 1% EDTA-free protease inhibitor cocktail, pH 8.5). This solution was applied again to a HisTrap column with binding buffer (50 mM Tris/HCl, 150 mM NaCl, 7 mM imidazole, pH 8.5), and the elution buffer (50 mM Tris/HCl, 150 mM NaCl, 0.5 M imidazole, pH 8.5) did not contain urea and contained a lower concentration of NaCl (150 mM). Eluted fractions containing enzyme were pooled, concentrated and rebuffered (25 mM Tris/HCl, 150 mM NaCl, pH 7) by using Vivaspin concentrators from Sartorius (MWCO 10,000).

2. MALDI-TOF mass spectra

2.1. GlcNAc–Trt 7

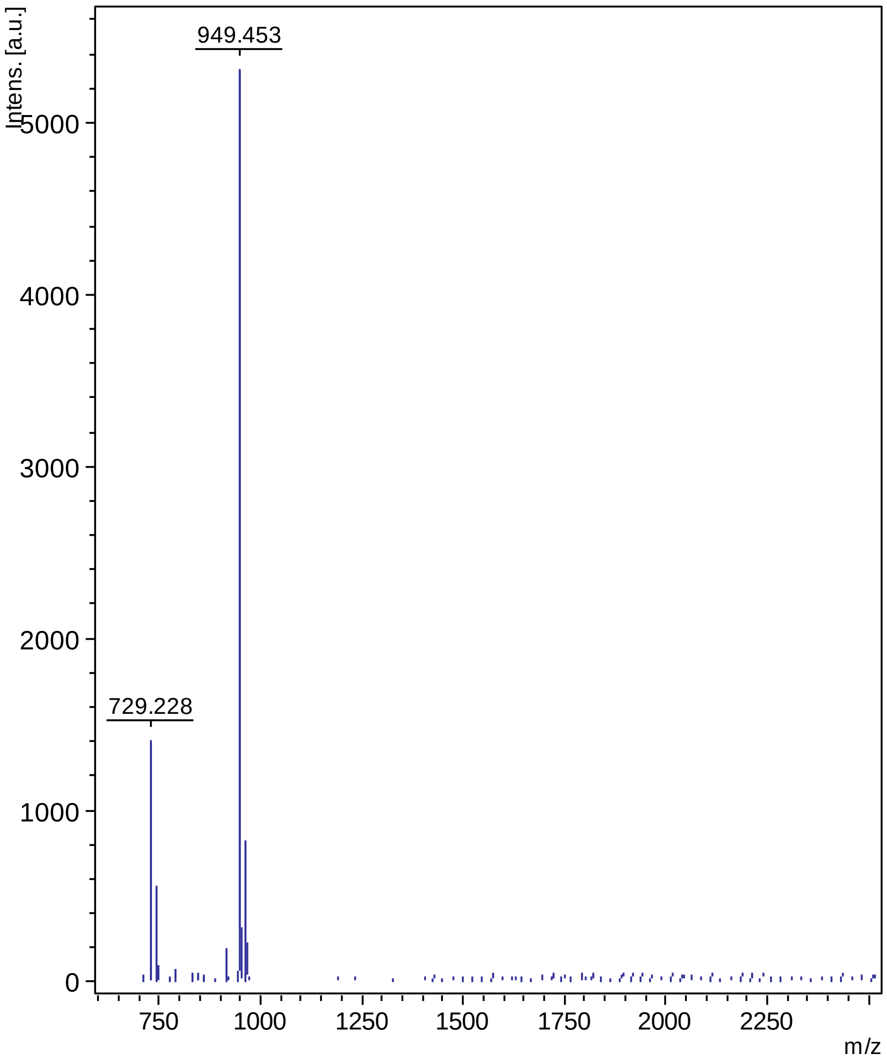

Figure S1: MALDI-TOF mass spectrum of GlcNAc–Trt **7** on aluminium-backed polystyrene support.

2.2. Polystyrene with aluminium strip

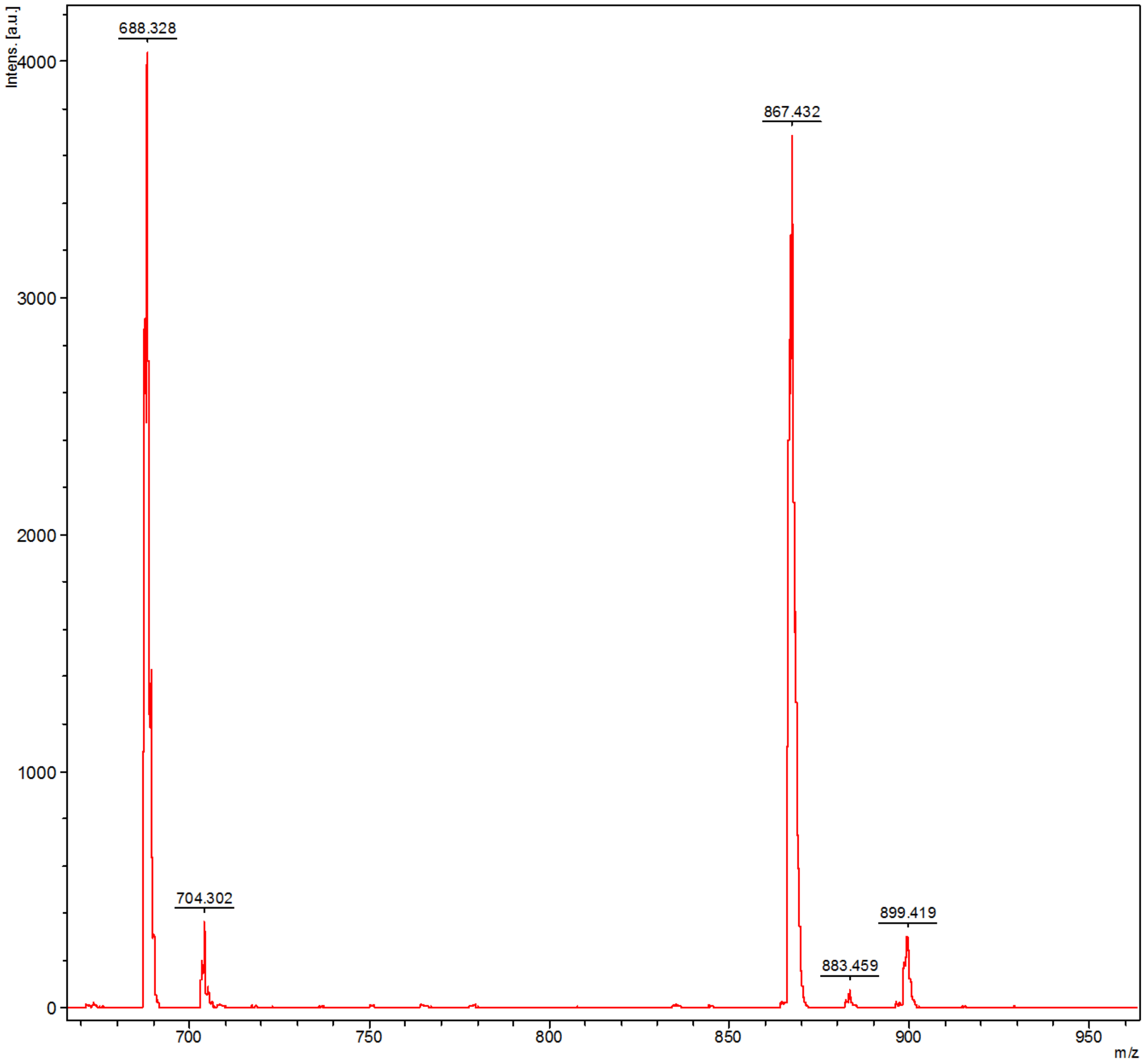

Figure S2: MALDI-TOF mass spectrum of Man–Trt **5** spotted away from the area where the aluminium strip is located.

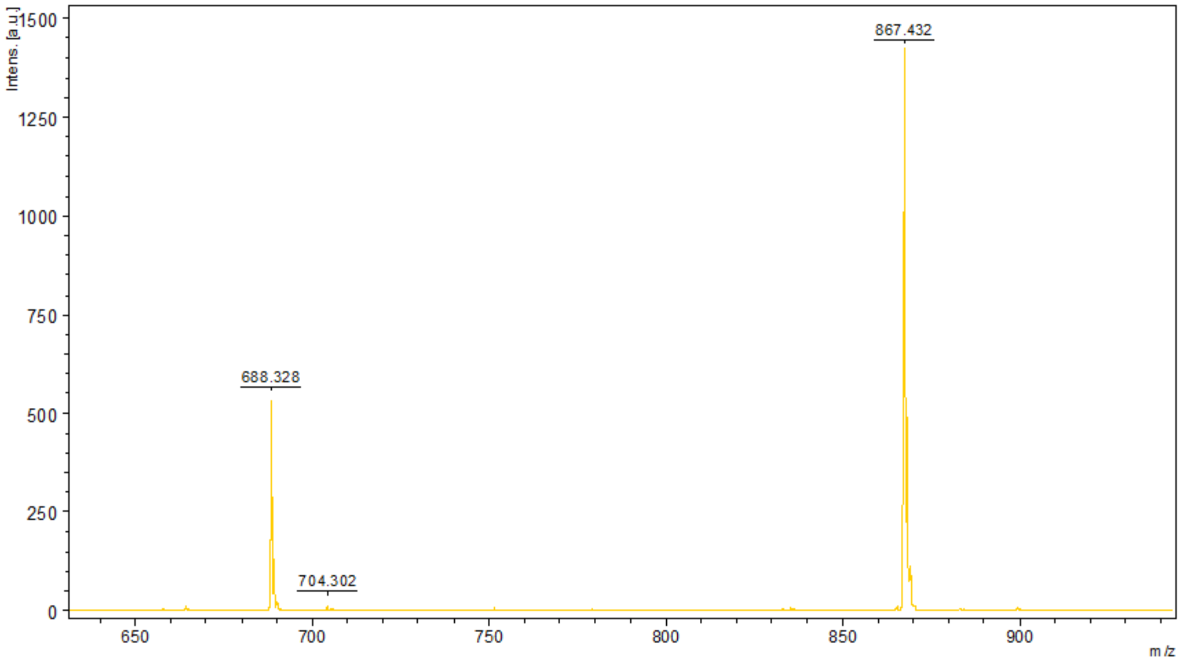

Figure S3: MALDI-TOF mass spectrum of Man–Trt **5** spotted over the area where the aluminium strip is located.

2.3. Polystyrene with aluminium rectangle

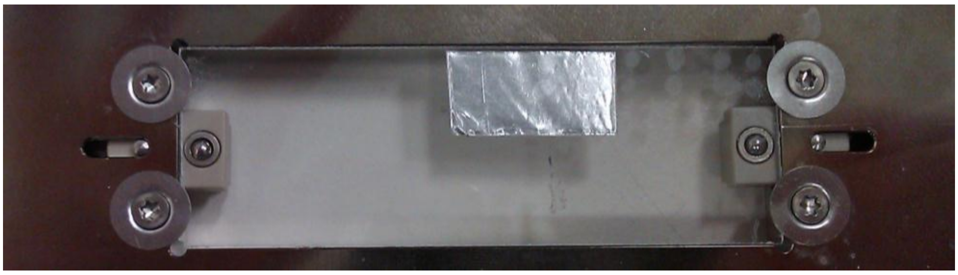

Figure S4: Photo of the aluminium rectangle backing. The aluminium rectangle is not in contact with the metal frame.

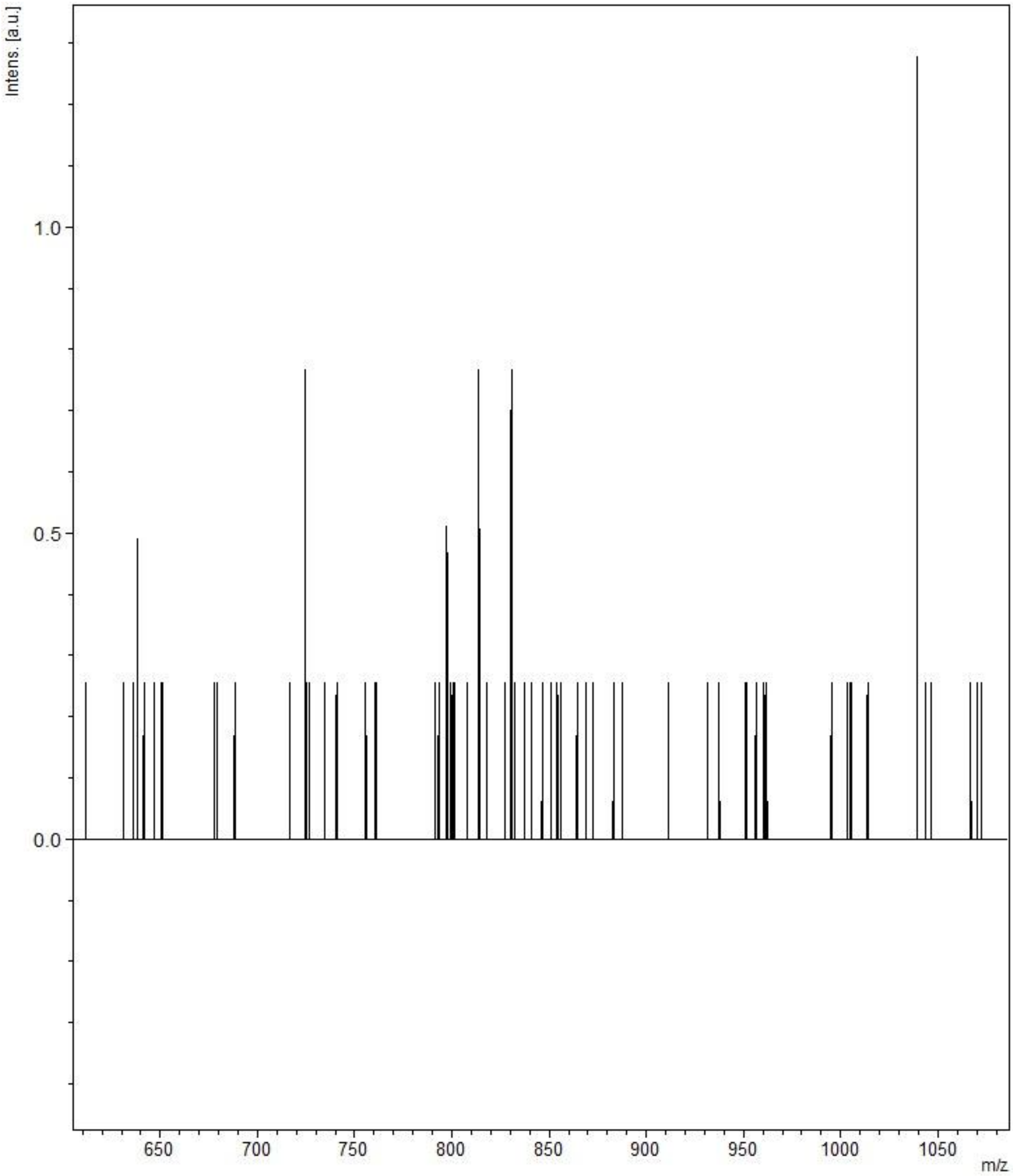

Figure S5: MALDI-TOF mass spectrum of Man–Trt **5** when only a rectangle of aluminium backing is applied, which does not make contact with the target support.

2.4. Limit of detection experiments

Serial dilutions of Man–Trt **5** linker were spotted onto steel, aluminium-backed polystyrene, and aluminium-backed glass plates to determine the limit of detection of the altered nonconductive plate in comparison to conventional steel target plates. In this case, the spots were not washed with water, so that the number of moles of linker in each spot would be more consistent.

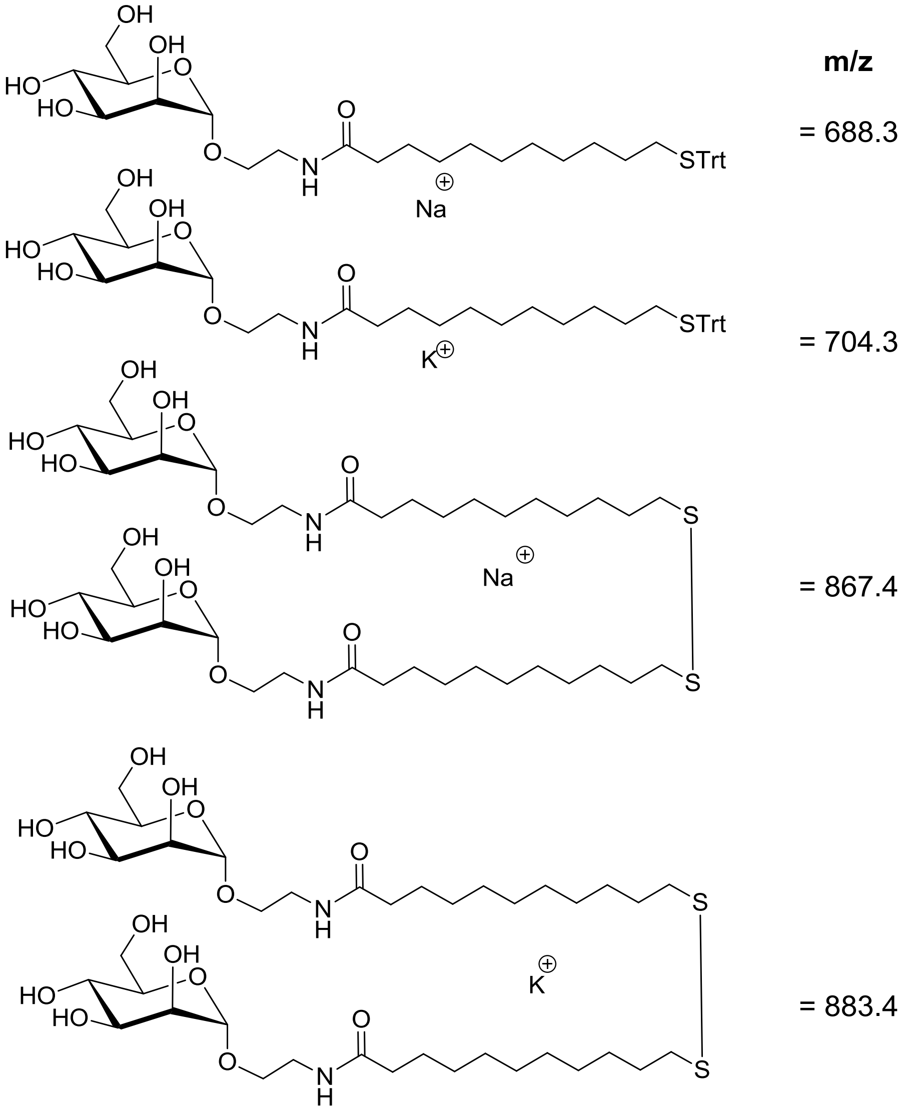

Figure S6: *m*/*z* of the observed ions in the mass spectrum of Man–Trt **5**.

2.4.1. Steel

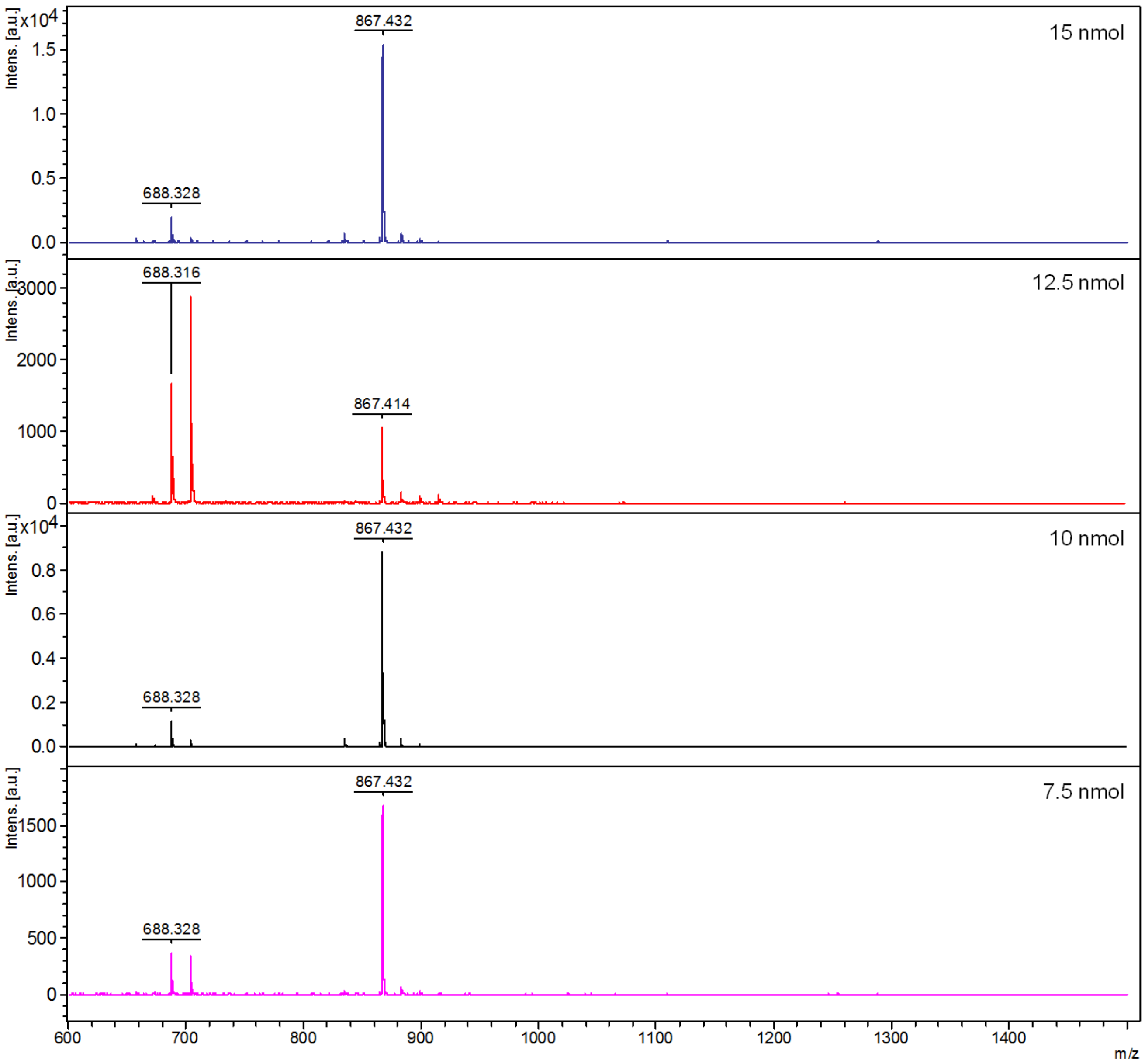

Figure S7: Serial dilution from 15 to 7.5 nmol of Man–Trt **5** analysed on a steel target plate.

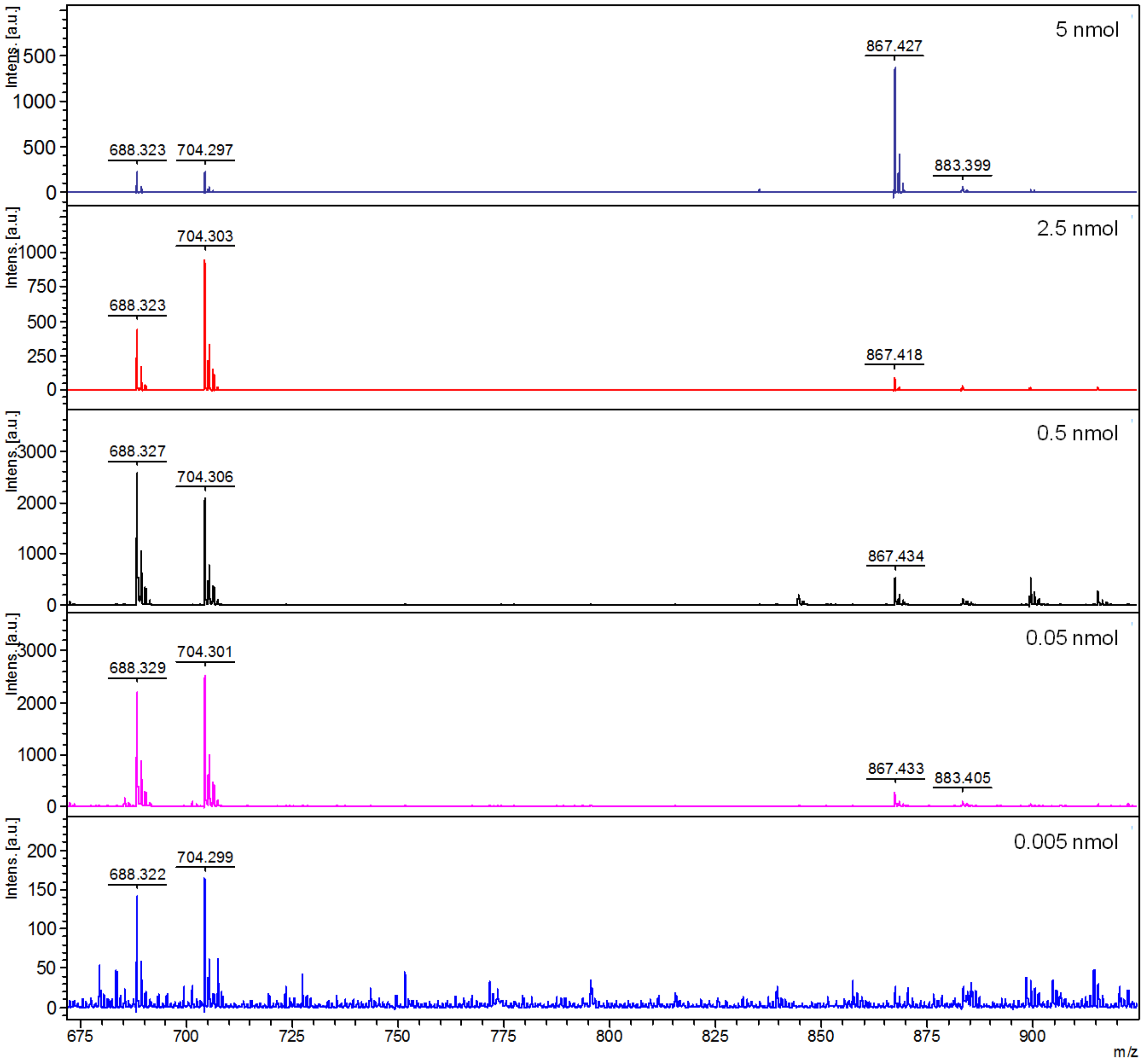

Figure S8: Serial dilution from 5 to 0.005 nmol of Man–Trt **5** analysed on a steel target plate.

2.4.2. Glass aluminium-backed

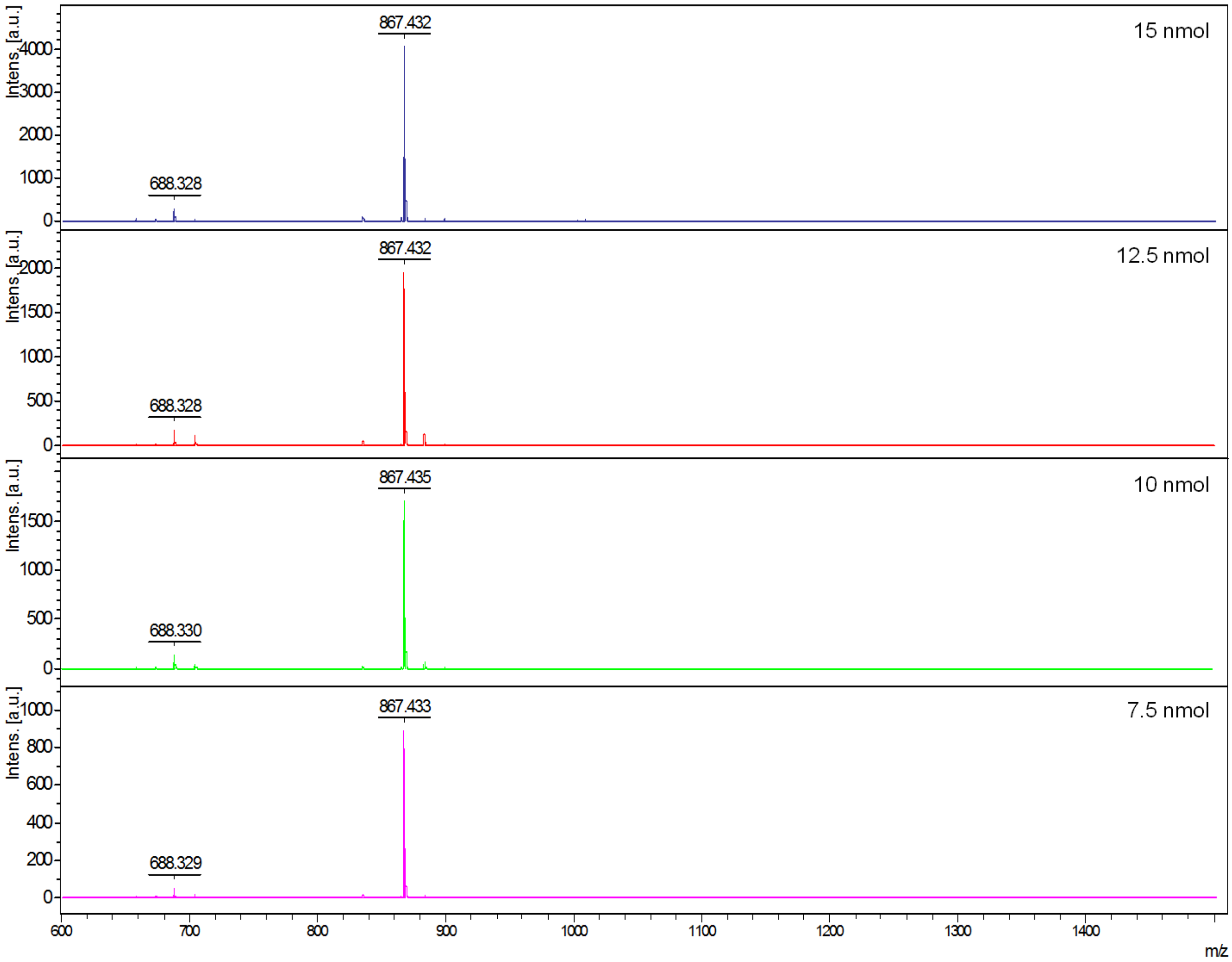

Figure S9: Serial dilution from 15 to 7.5 nmol of Man–Trt **5** analysed on an aluminium-backed glass slide.

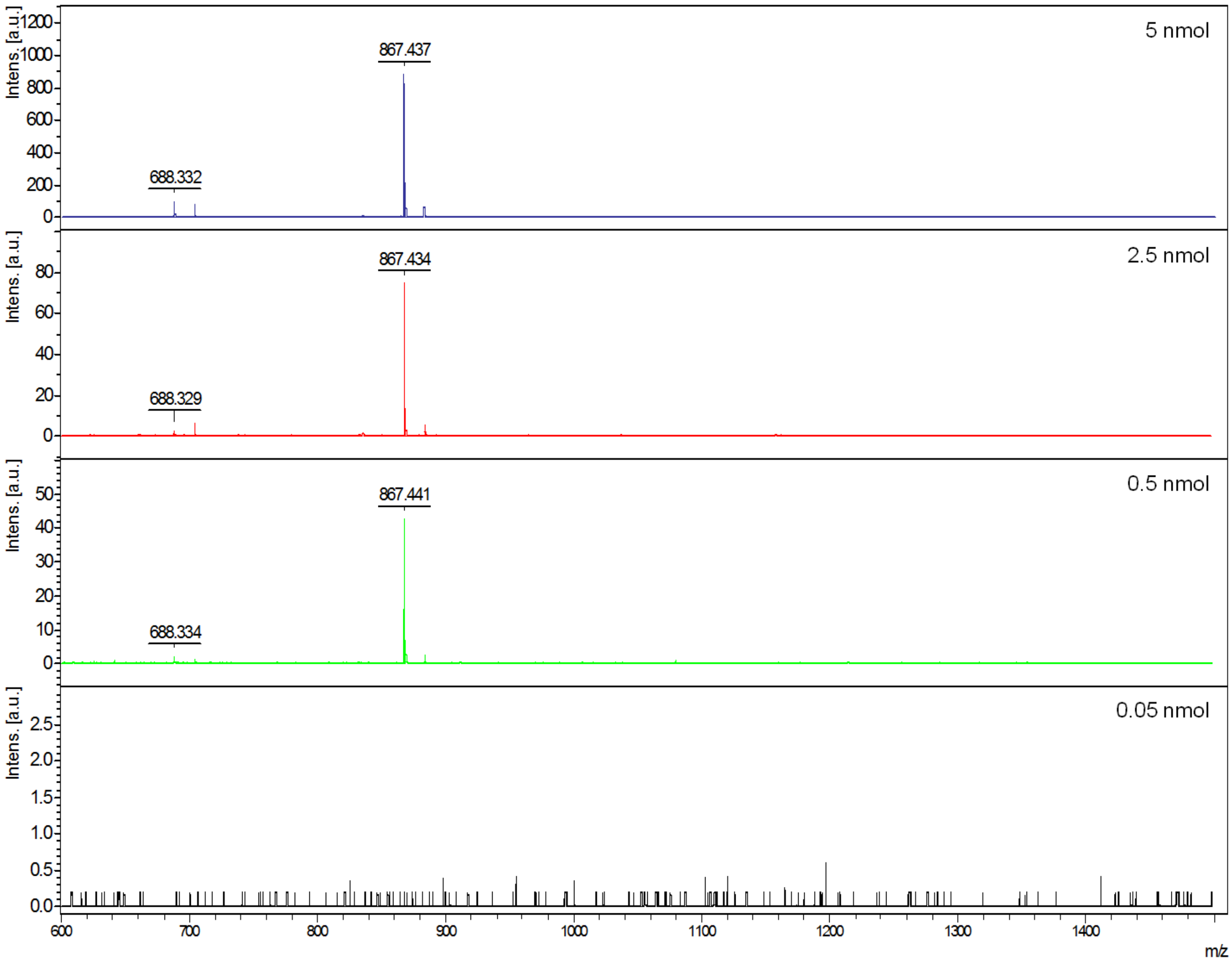

Figure S10: Serial dilution from 5 to 0.05 nmol of Man–Trt **5** analysed on an aluminium-backed glass slide.

2.4.3. Glass without aluminium backing

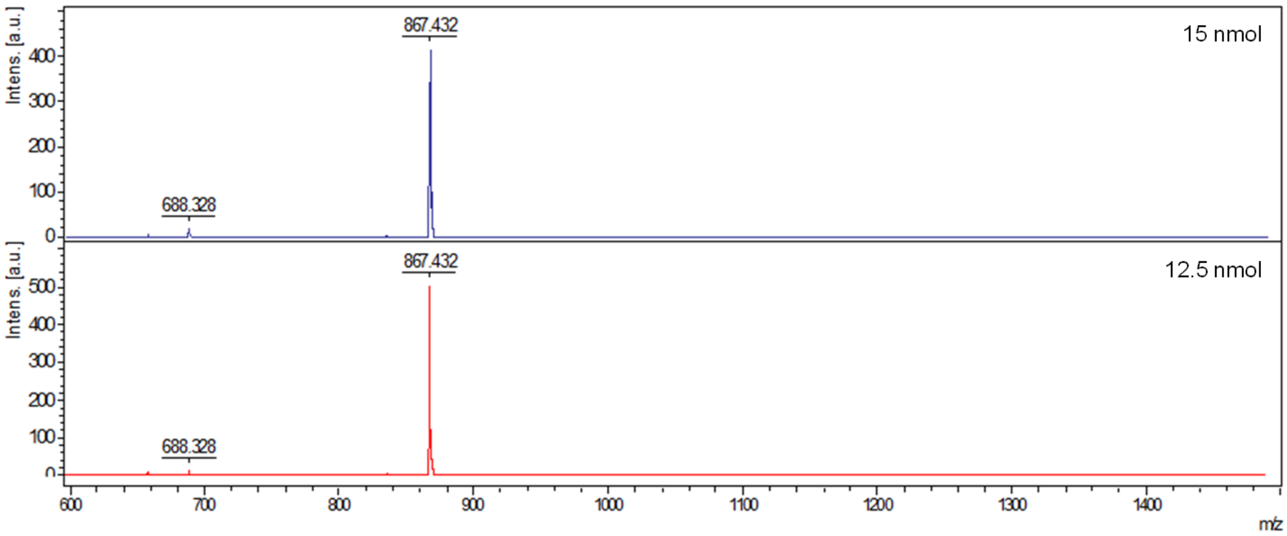

Figure S11: Serial dilution from 15 to 12.5 nmol of Man–Trt **5** analysed on a glass slide with no aluminium backing.

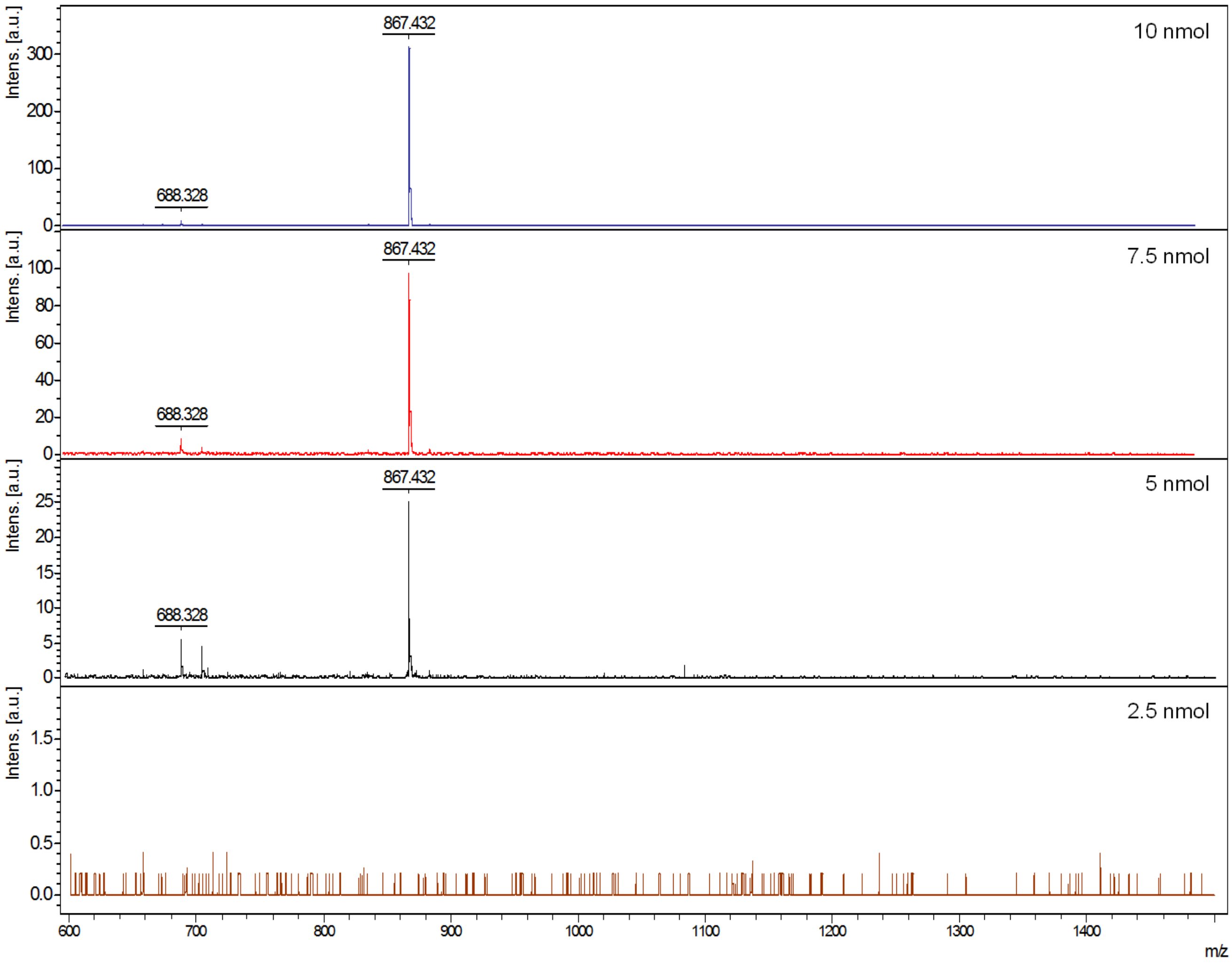

Figure S12: Serial dilution from 10 to 2.5 nmol of Man–Trt **5** analysed on a glass slide with no aluminium-backing.

2.4.4. Polystyrene aluminium-backed

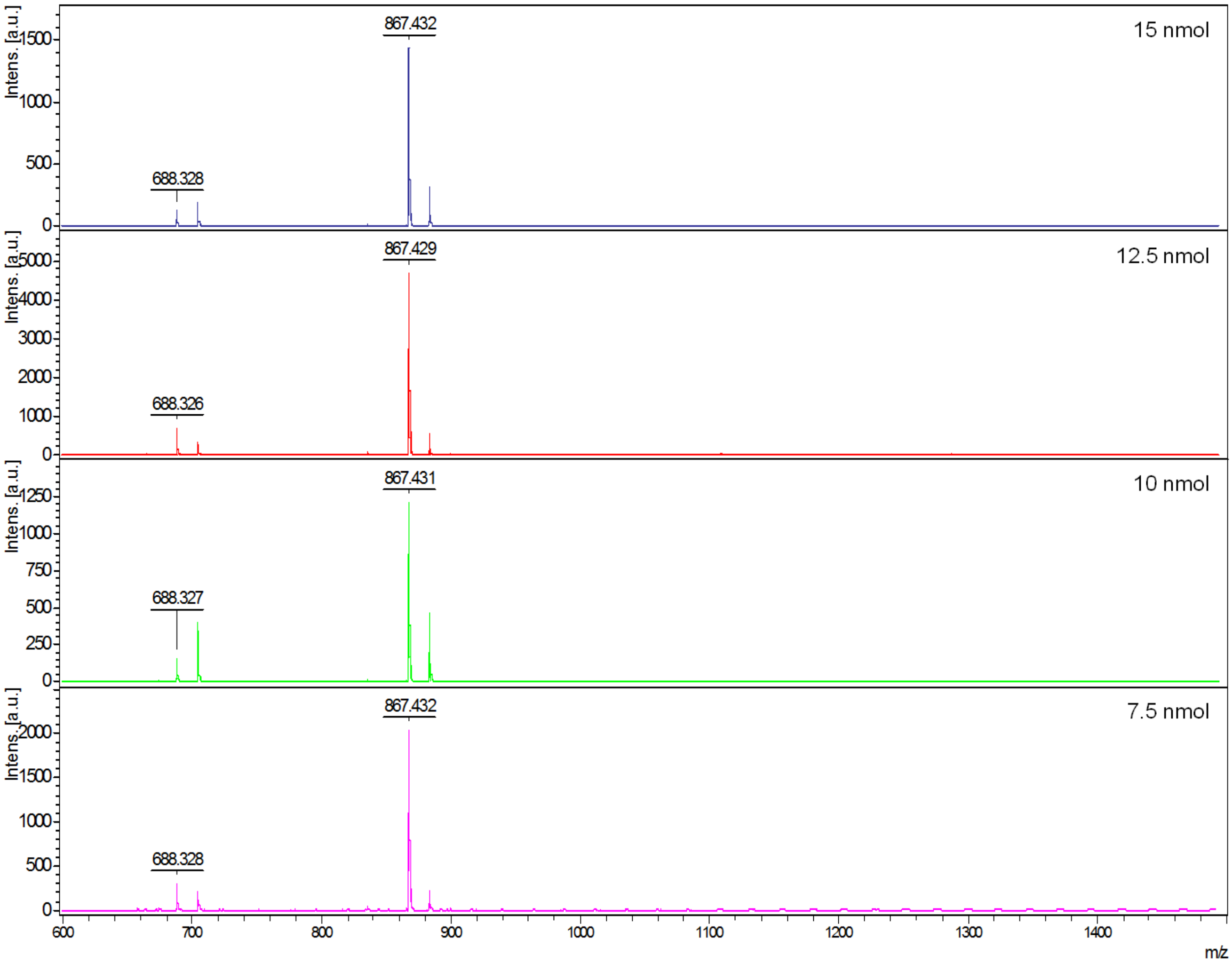

Figure S13: Serial dilution from 15 to 7.5 nmol of Man–Trt **5** analysed on aluminium-backed polystyrene plate.

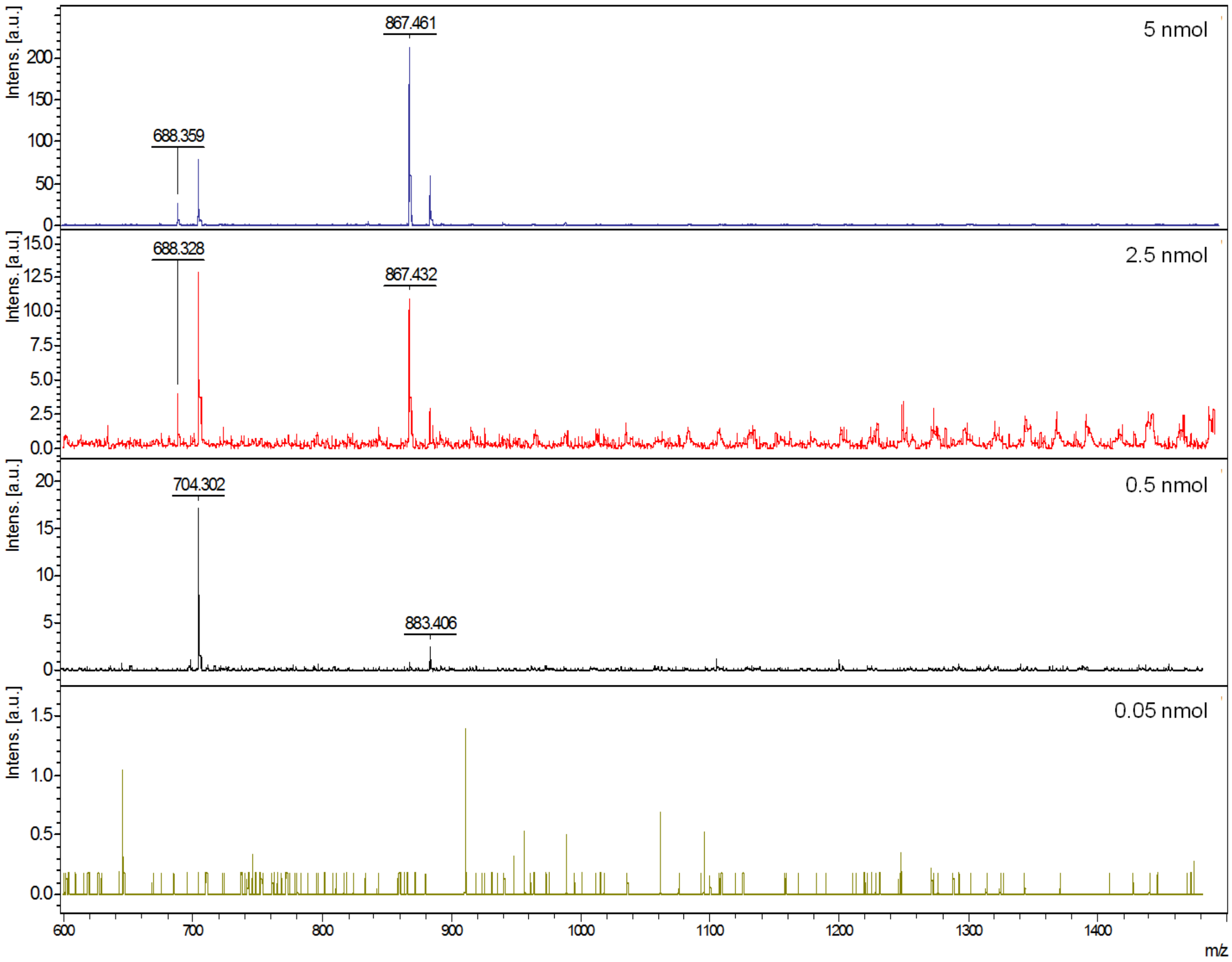

Figure S14: Serial dilution from 5 to 0.05 nmol of Man–Trt linker **5** analysed on aluminium-backed polystyrene plate.

As shown in Figure 2 of the main article, MALDI-TOF analysis of Man–Trt **5** on polystyrene showed no signal, and therefore there is no dilution series for the non-aluminium-backed support as there was in glass.
